# Development and validation of a one-step reverse transcription loop-mediated isothermal amplification (RT-LAMP) for rapid detection of ZIKV in patient samples from Brazil

**DOI:** 10.1038/s41598-021-83371-1

**Published:** 2021-02-18

**Authors:** Severino Jefferson Ribeiro da Silva, Keith Pardee, Udeni B. R. Balasuriya, Lindomar Pena

**Affiliations:** 1 Department of Virology, Aggeu Magalhães Institute (IAM), Oswaldo Cruz Foundation (Fiocruz), Avenida Professor Moraes Rego, Recife, Pernambuco 50670-420 Brazil; 2 Leslie Dan Faculty of Pharmacy, University of Toronto, Toronto, ON M5S 3M2 Canada; 3 Louisiana Animal Disease Diagnostic Laboratory and Department of Pathobiological Sciences, School of Veterinary Medicine, Louisiana State University, Baton Rouge, LA USA

**Keywords:** Virology, Assay systems

## Abstract

We have previously developed and validated a one-step assay based on reverse transcription loop-mediated isothermal amplification (RT-LAMP) for rapid detection of the Zika virus (ZIKV) from mosquito samples. Patient diagnosis of ZIKV is currently carried out in centralized laboratories using the reverse transcription-quantitative polymerase chain reaction (RT-qPCR), which, while the gold standard molecular method, has several drawbacks for use in remote and low-resource settings, such as high cost and the need of specialized equipment. Point-of-care (POC) diagnostic platforms have the potential to overcome these limitations, especially in low-resource countries where ZIKV is endemic. With this in mind, here we optimized and validated our RT-LAMP assay for rapid detection of ZIKV from patient samples. We found that the assay detected ZIKV from diverse sample types (serum, urine, saliva, and semen) in as little as 20 min, without RNA extraction. The RT-LAMP assay was highly specific and up to 100 times more sensitive than RT-qPCR. We then validated the assay using 100 patient serum samples collected from suspected cases of arbovirus infection in the state of Pernambuco, which was at the epicenter of the last Zika epidemic. Analysis of the results, in comparison to RT-qPCR, found that the ZIKV RT-LAMP assay provided sensitivity of 100%, specificity of 93.75%, and an overall accuracy of 95.00%. Taken together, the RT-LAMP assay provides a straightforward and inexpensive alternative for the diagnosis of ZIKV from patients and has the potential to increase diagnostic capacity in ZIKV-affected areas, particularly in low and middle-income countries.

## Introduction

Zika virus (ZIKV) is an enveloped virus belongs to the genus *Flavivirus* in the family *Flaviviridae*. Other notable viruses in this genus are West Nile virus (WNV), dengue virus (DENV 1–4), yellow fever virus (YFV), Japanese encephalitis virus (JEV), St. Louis encephalitis virus (SLEV), and Rocio virus (ROCV)^1–3^. The genome of ZIKV consists of a single-stranded positive-sense RNA molecule (+ ssRNA), with approximately 11 Kb in length and has a single open reading frame (ORF) which is flanked by 5′ and 3′ untranslated regions (UTRs), respectively^4^. ZIKV genome encodes three structural proteins [capsid (C), pre-membrane (prM) and envelope (E)] and seven non-structural proteins (NS1, NS2A, NS2B, NS3, NS4A, NS4B and NS5)^5^.

ZIKV was first isolated in 1947 during a yellow fever study in the Zika forest, Uganda^6^. In the 60 years that followed its discovery, few cases of ZIKV infection were reported in humans and the virus has remained an obscure pathogen. In April 2007, the ZIKV caused an outbreak of disease in Yap State, Federated States of Micronesia, and subsequently, spread rapidly across the Pacific causing outbreaks in French Polynesia, the Cook Islands, Easter Island, and New Caledonia^7–13^. The ZIKV was then introduced to Brazil, possibly in 2013, and in May 2015, the first case of ZIKV infection was reported in Brazil^14–16^. The virus then spread rapidly through the Americas and became a global health concern, causing the largest ZIKV epidemic recorded to date^14^. Besides Zika fever, the virus was responsible for a severe outbreak of microcephaly and other congenital abnormalities in neonates born to mothers infected during pregnancy, as well of many cases of neurological disorders such as Guillain–Barré syndrome (GBS) in infected patients^1,15,17–20^.

ZIKV is primarily transmitted to humans through the bites of infected female mosquitoes from the genus *Aedes*, mainly *Aedes aegypti* and *Aedes albopictus*, although other species of mosquitoes may also be involved in the transmission chain^21–27^*.* In addition to vector-borne transmission, ZIKV can be transmitted through sexual intercourse and blood transfusions, as well as during pregnancy^28–31^. In most patients, infection by ZIKV is asymptomatic and, when present, the clinical manifestation is characterized by the presence of fever, rash, myalgia, arthralgia and headache, which overlaps significantly with the features of DENV and chikungunya (CHIKV) infections^32^. Since these clinical symptoms are shared with infections from other arboviruses, diagnosing ZIKV infection using clinical indications alone is difficult. Therefore, accurate diagnostic platforms are important to identify and differentiate the etiologic agent of illness^1,33^.

In the clinical laboratory, ZIKV infection can be diagnosed using serology and molecular methods. Serological assays available include plaque reduction neutralization tests (PRNT), enzyme-linked immunosorbent assays (ELISA), and lateral flow assays to detect IgM or IgG antibodies^7,34^. However, cross reactivity with other flaviviruses, such as DENV, in serological assays limits their utility in regions where other flaviviruses circulate. Thus, reverse transcription-quantitative polymerase chain reaction (RT-qPCR) is considered the gold standard molecular method for ZIKV diagnosis and has been successfully use to detect ZIKV in serum, urine, saliva, semen and other body fluids^7,35–40^. Nevertheless, RT-qPCR is expensive, requires technical expertise, reliable access to electricity and utilizes sophisticated equipment for amplification and detection of viral genome. These drawbacks negatively impact the establishment of effective disease control programs caused by ZIKV, especially in low-resource settings. Moreover, the COVID-19 pandemic has worsened this situation in Brazil since most diagnostics resources have been directed for SARS-CoV-2 detection^41^.

Reverse transcription loop-mediated isothermal amplification (RT-LAMP) is a rapid, simple and robust tool for the rapid amplification of nucleic acid at a single and fixed temperature^42^. The assay has many advantages compared to other molecular methods including low cost, convenience, high sensitivity and specificity, making it a powerful method for point-of-care (POC) diagnostic^43^. Importantly, the isothermal nature of RT-LAMP reactions means that they can be performed without expensive equipment and, moreover, the colorimetric output means results can be determined with the naked eye^43^. Due to these advantages and potential for POC applications, many LAMP platforms for ZIKV have been developed since ZIKV emergence in the Americas^44^. However, many of these assays still require sophisticated equipment, expensive supplies or laborious protocols for ZIKV amplification and detection, which limits its applicability in low-resource settings.

We previously developed a one-step RT-LAMP assay for detection of ZIKV in mosquito samples that addressed the above-mentioned limitations of ZIKV LAMP assays^45^. Our strategy is based on a close-tube, one-step protocol and does not require RNA extraction from the biological specimens. With these features in-place, our main goal here was we develop and validate a one-step RT-LAMP assay for ZIKV detection in different human specimens, including serum, urine, saliva, and semen. Detection of the virus was achieved in as little as 20 min without RNA extraction or any pretreatment of the patient samples. We then validated the performance of this assay for using patient samples collected in Pernambuco State, Brazil, which was the epicenter of the last ZIKV epidemic. The results validated our assay for POC applications for human diagnosis and does not require expensive equipment and can be performed by an operator with minimal technological expertise. This RT-LAMP technology represents a rapid, sensitive, and specific assay for ZIKV diagnosis and has the potential to improve diagnostic capabilities and surveillance actions in remote areas and countries with limited laboratory infrastructure.

## Results

### Optimization of ZIKV RT-LAMP reaction conditions

**Figure 1:**
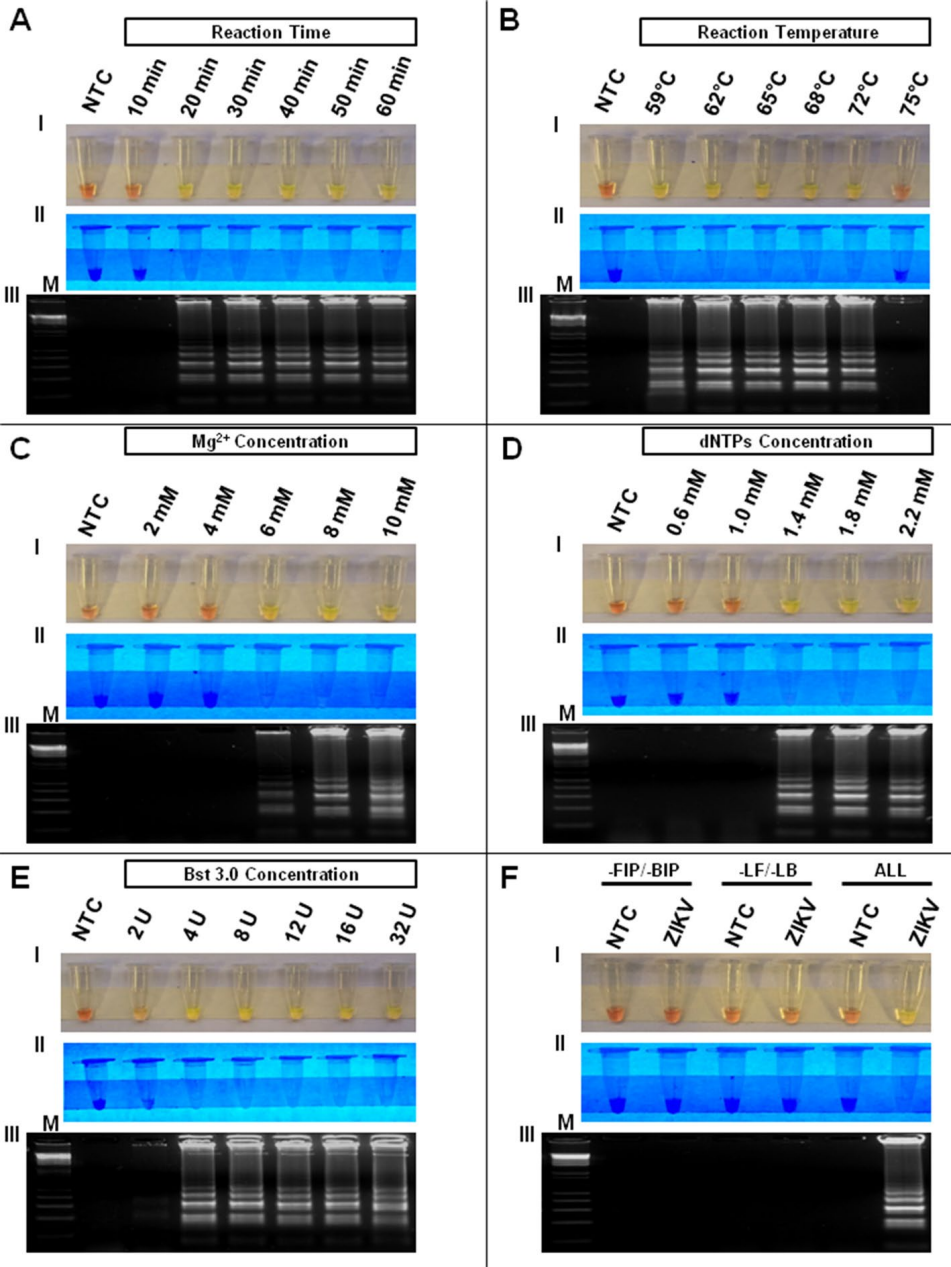
Optimization of reagent concentrations and conditions of the ZIKV RT-LAMP assay. All reagent concentrations as well as all RT-LAMP assay conditions were optimized and established. The reactions were performed under incubation time ranging from 10 to 60 min (**A**) and temperatures ranging from 59 to 75 °C (**B**). The reagents were optimized for optimal concentrations of Mg^2+^ (ranging from 2 to 10 mM) (**C**), dNTPs (ranging from 0.6 to 2.2 mM) (**D**) and Bst 3.0 DNA polymerase (ranging from 2 to 32 U) (**E**). In addition, all primers, including external primers (F3 and B3), internal (FIP and BIP) and loop (LF and LB) were required to perform the RT-LAMP assay (**F**). The RT-LAMP results were observed by visual color change of the products in the reaction tube and by gel electrophoresis. Amplification products were observed by naked eye natural light (A-FI) under UV irradiation (A-FII) and agarose gel electrophoresis (A-FIII). Legends: NTC (non-template control): water; M: molecular weight marker. Orange color: negative reaction for ZIKV; Greenish yellow color: positive reaction for ZIKV.

To optimize ZIKV RT-LAMP assay performance for human samples, we screened reaction conditions such as temperature, incubation time, Mg^2+^ concentration, dNTPs concentration, enzyme concentration and primer sequences. We also evaluated whether all primers, including the external (F3 and B3), internal (FIP and BIP) and loop (LF and LB) primers were required for successful amplification of target RNA. Initially, we performed a time course series and found that ZIKV genome amplification (10^5^ PFU) occurred with incubation time as short as 20 min (Fig. 1A), but 40 min of incubation provided more consistent results for low titer samples. Positive amplification was detected at incubation temperatures ranging from 59 to 72 °C (Fig. 1B). Based on these results, we carried out the other assays at the temperature of 72 °C for 40 min to maximize the chance of virus detection (sensitivity) and the specificity, respectively. Based on reagent titrations, the optimal concentrations for the Mg^2+^, dNTPs, and Bst 3.0 DNA polymerase were determined to be 8 mM, 1.8 mM, and 4U of the enzyme, respectively (Fig. 1C–E). All six primers were required for amplification of the RNA target, since removal of either backward and forward inner (FIP/BIP) or loop primers (LF/LB) failed to produce a positive reaction (Fig. 1F). These optimized parameters were used for subsequent experiments described below.

### Detection of ZIKV in virus-spiked human samples

**Figure 2:**
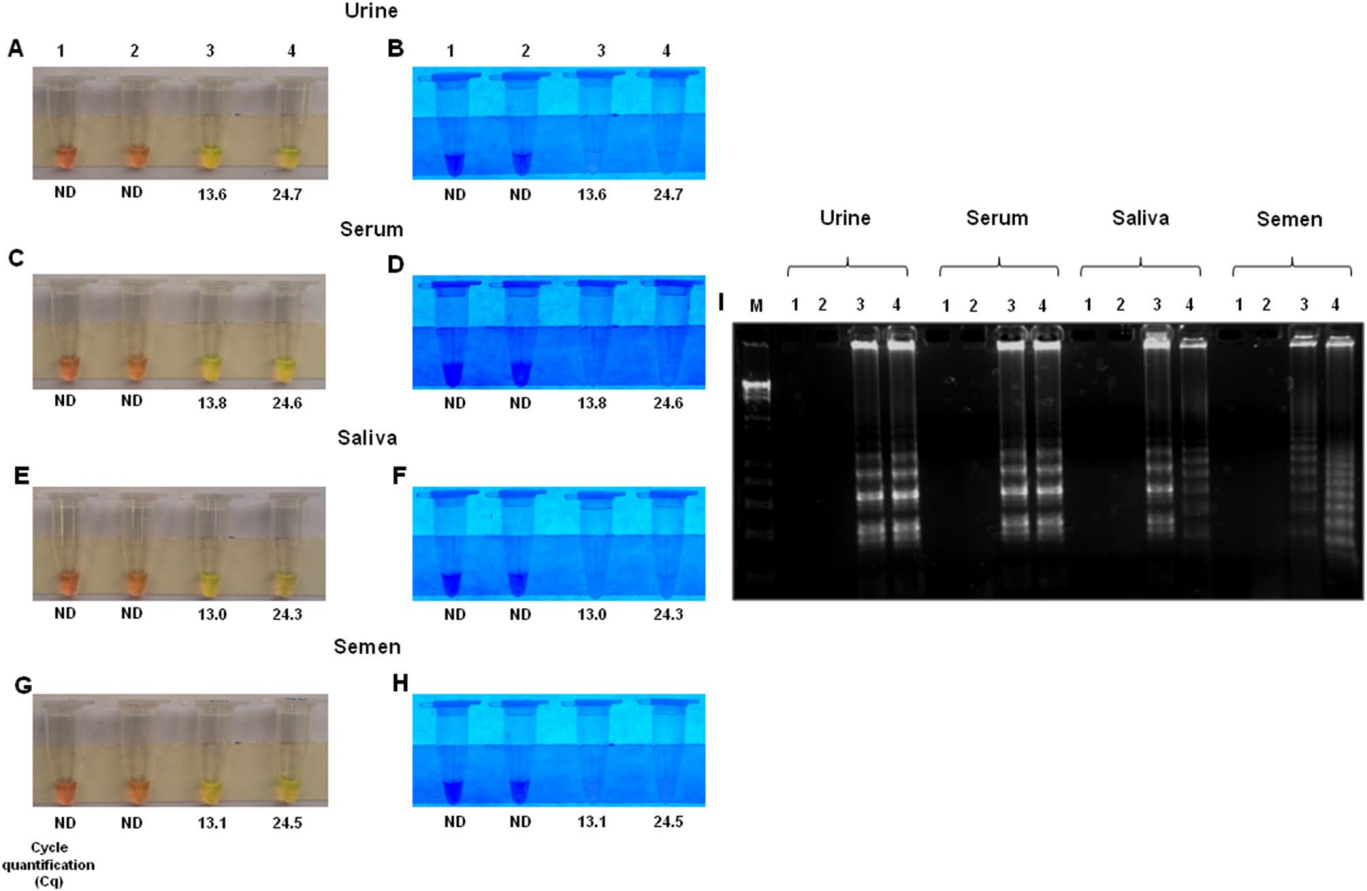
Detection of ZIKV in virus-spiked body fluid samples. Biological samples including urine, serum, saliva and semen were spiked with either a high (1 × 10^6^ PFU/mL) or low viral load (1 × 10^3^ PFU/mL) and processed for RT-LAMP without RNA extraction (**A**–**I**). The RT-LAMP results were observed by visual color change of the products in the reaction tube and by gel electrophoresis. Amplification products were observed by naked eye natural light (**A**, **C**, **E** and **G**), under UV irradiation (**B**, **D**, **F** and **H**) and agarose gel electrophoresis (I). Legends in (**A**–**I**) are: (1) NTC (non-template control): water; (2): uninfected biological sample (urine, serum, saliva and semen); (3): biological sample infected with 10^6^ PFU/mL; (4): biological sample infected with 10^3^ PFU/mL. M: molecular weight marker. Orange color: negative reaction for ZIKV; Greenish yellow color: positive reaction for ZIKV.

To evaluate the ability of the RT-LAMP assay to detect ZIKV in clinically relevant specimens, we spiked urine, serum, saliva and semen obtained from healthy, consenting, adult volunteers with a high viral load (1 × 10^6^ PFU/mL) followed by a 1:1000 dilution in water to achieve a low viral load (1 × 10^3^ PFU/mL). Samples were infected for 1 h at 37 °C and then directly assayed by RT-LAMP assay without RNA extraction using the same volume of human biofluids. ZIKV was detected in all spiked samples, regardless of the viral dose. In semen samples, the high and low viral load gave similar results as detected by naked eye and UV irradiation, but the low viral load spike gave better amplification then the high viral load as detected by agarose gel analysis. As expected, non-template control (NTC) samples (water) and negative control (human biological sample uninfected) tested negative (Fig. 2A–I). RT-LAMP results were compared to RT-qPCR, through which the cycle quantification (Cq) values^46^ were (13.6; 13.8; 13.0; 13.1) and (24.7; 24.6; 24.3; 24.5), for high viral and low viral load in urine, serum, saliva and semen, respectively.

### Analytical specificity of ZIKV RT-LAMP assay

**Figure 3:**
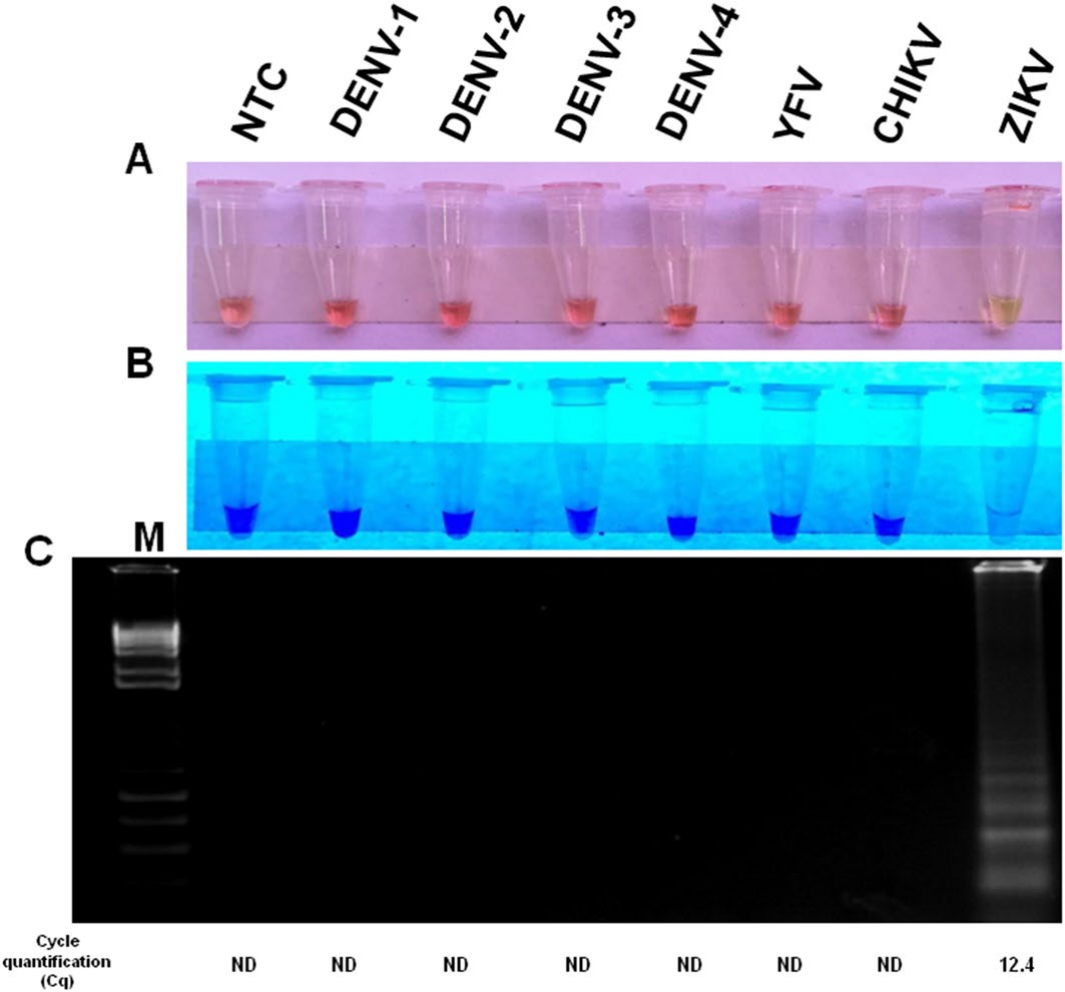
Analytical specificity of ZIKV RT-LAMP in human serum. Human serum sample uninfected was spiked with different arboviruses (DENV 1–4, YFV, CHIKV, ZIKV) circulating in Brazil and then assayed by ZIKV RT-LAMP without RNA extraction. The amplification products were observed by naked eye under natural light (**A**), under UV irradiation (**B**) and agarose gel electrophoresis (**C**). NTC (non-template control): water. M: molecular weight marker. Orange color: negative reaction for ZIKV; Greenish yellow color: positive reaction for ZIKV.

In order to evaluate the specificity of the RT-LAMP primers to detect only ZIKV, we assayed human serum spiked with key endemic arboviruses circulating in Brazil, including DENV-1 (PE/97-42735), DENV-2 (PE/95-3808), DENV-3 (PE/02-95016), DENV-4 (PE/10-0081), YFV (17DD), and CHIKV (PE2016-480) (Table 1). RT-LAMP specifically detected ZIKV as determined by naked eye analysis, visual observation under UV light or agarose gel electrophoresis (Fig. 3). These results were confirmed by RT-qPCR, in which the Cq value for the ZIKV sample was 12.4 (Fig. 3). A common problem with highly sensitive RT-LAMP assays is cross-contamination. To prevent this, we added 1 μL of 1:10 dilution of SYBR Green I dye diluted in RNase-free water to the center of the tube lid caps before the reaction and mixed afterwards^47^. This reduces the potential for the introduction of contamination and in work performed here, no contamination was seen observed using the one step, closed tubes RT-LAMP strategy (data not shown).

**Table 1:** Viruses used to evaluate the analytical specificity of the ZIKV RT-LAMP assay.

Viruses	Strain	GenBank access code
*Flaviviridae*, *flavivirus*, Zika virus	PE243	KX197192
*Flaviviridae*, *flavivirus*, dengue virus serotype 1	PE/97-42735	EU259529
*Flaviviridae*, *flavivirus*, dengue virus serotype 2	PE/95-3808	EU259569
*Flaviviridae*, *flavivirus*, dengue virus serotype 3	PE/02-95016	KC425219
*Flaviviridae, flavivirus*, dengue virus serotype 4	PE/10-0081	Unpublished
*Flaviviridae*, *flavivirus*, yellow fever virus	17DD	DQ100292
*Togaviridae*, *alphavirus*, chikungunya virus	PE2016-480	Unpublished

### Analytical sensitivity of ZIKV RT-LAMP assay

**Figure 4:**
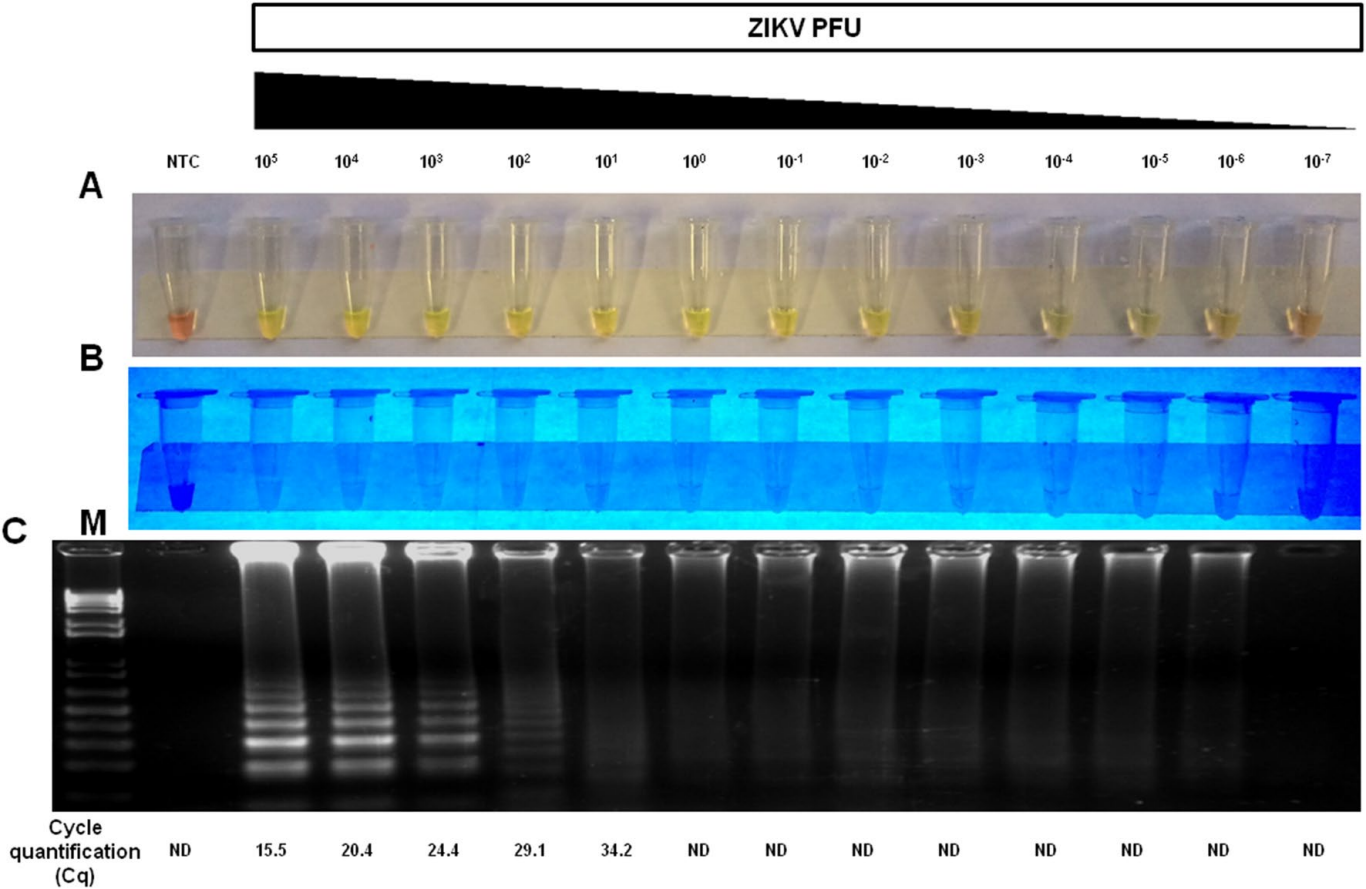
Analytical sensitivity of ZIKV RT-LAMP in human serum. The sensitivity of RT-LAMP was determined based on the last dilution at which the viral genome was detected by RT-LAMP assay. The sensitivity of RT-LAMP was determined by spiking different amounts (10^5^ to 10^–7^ PFU) of ZIKV in human serum sample uninfected and then testing by RT-LAMP directly without RNA extraction. The amplification products were observed by naked eye under natural light (**A**), under UV irradiation (**B**), agarose gel electrophoresis from RT-LAMP (**C**) and agarose gel electrophoresis from RT-PCR. M: molecular weight marker. NTC (non-template control): water. ND (Not detected). To compare the results of RT-LAMP with RT-qPCR, viral RNA was extracted from the same dilutions and assayed. Orange color: negative reaction for ZIKV; Greenish yellow color: positive reaction for ZIKV.

To evaluate the analytical sensitivity (limit of detection—LOD) of the assay, RT-LAMP was performed in human serum spiked with varying concentrations (10^5^ PFU to 10^–7^ PFU) of ZIKV. Spiked samples were directly assayed by RT-LAMP without RNA extraction. RT-LAMP was able to detect a broad range of virus concentration (from 10^5^ to 10^−6^ PFU). Then, viral RNA of same dilutions were extracted and assayed by the gold standard RT-qPCR test. The analytical sensitivity of RT-qPCR was only observed down to 10^1^ PFU with a Cq value of 34.2 (Fig. 4). The experiments were independently repeated 10 times to allow probit regression analysis to accurately determine the limit of detection of RT-LAMP. The limit of detection of RT-LAMP at 95% probability was − 1.07 log_10_ PFU of ZIKV with confidence interval from − 1.93 to 0.49 (Table 2 and Fig. S5), which is 100-fold more sensitive than RT-qPCR for ZIKV. Similar analytical sensitivity results were also obtained in urine, saliva and semen (data not shown).

**Table 2:** Limit of detection (LOD) of the RT-LAMP assay for ZIKV detection.

ZIKV concentration (PFU)	Number of replicates	Number of positive results	Hit rate in %
10^5^	10	10	100
10^4^	10	10	100
10^3^	10	10	100
10^2^	10	10	100
10^1^	10	10	100
10^0^	10	10	100
10^–1^	10	9	90
10^–2^	10	9	90
10^–3^	10	7	70
10^–4^	10	4	40
10^–5^	10	3	30
10^–6^	10	1	10
10^–7^	10	0	0

### Diagnostic performance and cost of the RT-LAMP assay for detection of ZIKV in patient samples

**Figure 5:**
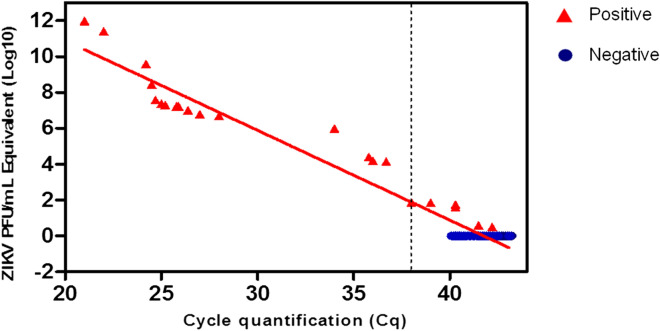
Diagnostic of human samples by RT-LAMP. A total of 100 serum human samples from were tested for ZIKV by RT-LAMP assay. Of these, 20 were positive for ZIKV and 80 were negative as determined by RT-qPCR. Dashed line represents the RT-qPCR Cq threshold for ZIKV positivity (Cq ≤ 38). Red triangle indicates samples positive by RT-LAMP and blue circle are samples negative by RT-LAMP assay.

We next validated the RT-LAMP assay using clinical samples obtained from patients with suspected arbovirus infection. A total of 100 serum samples double blinded (20 positive and 80 negative for ZIKV by RT-qPCR) (Table S1) were used in the experiment. The Cq value in these samples ranged from 21.0 to > 40.0 and samples with Cq values of ≤ 38.0 in triplicate wells were considered positive for ZIKV by RT-qPCR. The RT-LAMP assay detected ZIKV in 25 samples, including five samples which had been determined negative by gold standard method, whereas 75 were deemed negative by ZIKV RT-LAMP (Fig. 5).

The diagnostic performance of this assay for detection of ZIKV was inferred by statistical analysis of several parameters using RT-qPCR assay as reference test. The RT-LAMP assay had a clinical sensitivity of 100% (95% CI 83.16% to 100.00%) and clinical specificity of 93.75% (95% CI 86.01% to 97.94%). The overall ZIKV prevalence in the samples was 20.00% (95% CI 12.67% to 29.18%). The positive predictive value of this assay was 80.00% (95% CI 63.13% to 90.33%) and negative predictive value was 100%. The overall accuracy of the ZIKV RT-LAMP assay was 95.00% (95% CI 88.72% to 98.36%) (Table 3), highlighting the diagnostic positive features of the RT-LAMP assay for rapid detection of ZIKV in patient samples.

**Table 3:** Diagnostic performance of RT-LAMP assay for ZIKV detection in patient samples.

	RT-qPCR+	RT-qPCR−	Total
RT-LAMP+	20	5	25
RT-LAMP−	0	75	75
Total	20	80	
Sensitivity	**100%** (95% CI 83.16% to 100.00%)		
Specificity	**93.75%** (95% CI 86.01% to 97.94%)		
ZIKV prevalence	**20.00%** (95% CI 12.67% to 29.18%)		
Positive predictive value	**80.00**% (95% CI 63.13% to 90.33%)		
Negative predictive value	**100%**		
Accuracy	**95.00%** (95% CI 88.72% to 98.36%)		

**Figure 6:**
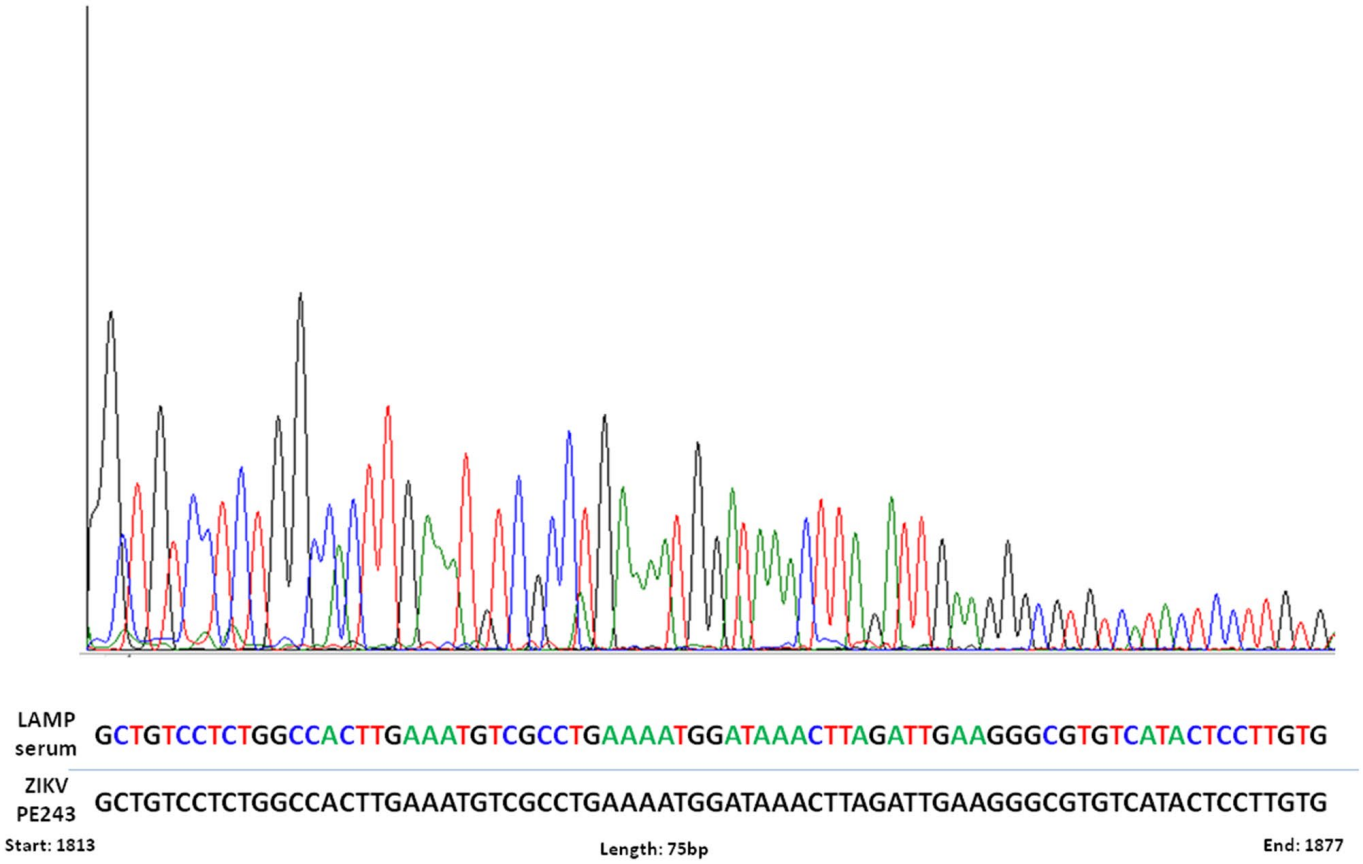
Electropherogram of ZIKV RT-LAMP detected in human serum. RT-LAMP amplicons from human serum was sequenced using Sanger method to confirm the identity of ZIKV. The region amplified was genome position 1813 to 1877. The obtained sequences were aligned against the ZIKV PE243 reference strain.

To confirm the identity of positive samples by RT-LAMP for ZIKV detection in human samples, we sequenced a positive sample of human serum by the Sanger method. Analyzes obtained from the sequenced amplicons and BLAST analysis demonstrated that ZIKV RT-LAMP amplicons match 100% with virus circulating in Brazil (Fig. 6), proving the specificity of the RT-LAMP for detection only ZIKV. Together, these results suggested that RT-LAMP protocol described here is highly specificity for detection of ZIKV.

We then compared the costs for consumables required RT-LAMP (Table S2) with RT-qPCR (Table S3) considering the prices (in US$) for reagent acquisition in Brazil at the time of the study. The estimated reagent cost for our RT-LAMP assay was US$ 0.32 as opposed to US$ 10.33 for RT-qPCR, which makes the RT-LAMP 33 times cheaper than the gold-standard method.

## Discussion

Given the rapid spread of ZIKV in the Americas and its association with increased incidence of congenital defects in newborns and Guillain-Barré syndrome in infected patients, reliable and fast POC assays for accessible and inexpensive ZIKV diagnosis are urgently needed^44^. In areas with past or active ZIKV circulation such as Brazil, monitoring virus activity and disease incidence is critical to allow timely interventions and avoid massive outbreaks^48^. Moreover, early identification of virus infection directly in the field after patient sample collection is a critical step to containing the spread of ZIKV^49^. Despite its emergence in Brazil in 2015, the country still relies on expensive RT-qPCR testing for diagnosing ZIKV infection. The availability of testing for ZIKV has been even more challenging during the COVID-19 pandemic since most diagnostic supplies has been directed for SARS-CoV-2 detection.

The clinical diagnosis of ZIKV infection based on clinical symptoms alone is very difficult in countries where other arboviruses are endemic, and definitive confirmation is requires laboratory testing. Currently, RT-qPCR is considered the gold standard molecular method for lab-based diagnosis of ZIKV, but the technique has several limitations that preclude its wide use in remote or low-resource settings. Here, we developed a one-step closed tube RT-LAMP assay for ZIKV diagnosis that can be deployed in low-resource settings. The assay is rapid, specific, affordable, and allows straightforward detection of ZIKV in human samples, including serum, urine, saliva and semen even in the absence of RNA extraction or sample pre-treatment.

Serological assays are another alternative for laboratory ZIKV diagnosis, but lack specificity due to antibody cross-reactivity with other flaviviruses, especially DENV^7,15,50,51^. Here, our ZIKV RT-LAMP assay showed no cross-reactivity with other arboviruses including CHIKV, YFV and DENV 1–4 (Fig. 3). Additional confirmation of specificity was provided by Sanger sequencing of RT-LAMP amplicons, which confirmed 100% match with ZIKV circulating in Brazil (Fig. 6).

Since the emergence of ZIKV in the Western hemisphere, many LAMP assays have been developed for the diagnosis of ZIKV by research groups across the world, including one-step and two-step procedures^44^. The two-step LAMP protocol requires the addition of a reverse transcriptase (RT) enzyme together with the DNA polymerase for amplification and detection of the viral genome. In addition, the two-step protocol needed longer times, requires additional samples and reagents handling, which increases the probability pipetting errors and contamination^44,45^. In order to overcome these drawbacks of two-step protocol, we used the Bst 3.0 DNA polymerase 3.0 WarmStart due to the fact that it is an enzyme that allows the assay to be performed in a one-step protocol without RNA extraction or pre-treatment of the sample. This is possible because Bst 3.0 enzyme has reverse transcriptase and DNA polymerase activities at fixed temperature incubation. Moreover, this enzyme maintains its performance even in the presence of amplification inhibitors and remains stable at room temperature compared to other prototypes of Bst enzyme (wild-type Bst DNA polymerase or Bst 2.0 DNA polymerase)^44,52^. Despite their resistance to the presence of inhibitors, we found that samples with high semen concentration seemed to be have an effect on target amplification (Fig. 2I) which may be due to the presence of inhibitors in semen which are known to affect other DNA polymerases^53^.

Another potential limitation of molecular methods is to detect low viral loads in ZIKV-infected that samples usually have low titers after the acute phase of ZIKV infection. This makes it very difficult to confirm ZIKV infection in patient samples, even using the RT-qPCR^54^. Wang et al*.* developed a diagnostic test based on RT-LAMP for detection of ZIKV in human samples. The limit of detection of this assay was determined to be 0.02 PFU/mL using ZIKV-spiked samples including serum, urine, and saliva^55^. Tian et al. also reported a assay based on RT-LAMP combined with AC susceptometry in a portable reaction and LOD was determined to be 1 aM (aM: 10^−18^ mol per liter) using human serum spiked with synthetically oligonucleotides of ZIKV^52^. These assays were able to detect low ZIKV concentrations, but the experiments and evaluation of the assay performance were conducted only with spiked samples, which not correlate with viral load presents in clinical samples obtained from infected patients.

Moreover, several studies based on the RT-LAMP assay have reported that the analytical sensitivity (limit of detection—LOD) is lower or similar when compared to RT-qPCR^54, 55^. In contrast, we developed a RT-LAMP assay for detection of ZIKV in mosquito samples from Brazil, which was able to detect up to 10^–5^ PFU. This represented a 10,000-fold greater sensitivity compared with RT-qPCR^45^. In human samples, our assay was 100-fold more sensitive than RT-qPCR. In the latter work, the virus was diluted in serum whereas in the former paper the virus was spiked in mosquito lysate and then serially diluted in water before the RT-LAMP. The reasons for the decreased sensitivity of the assay in human samples compared to mosquito samples are not clear, but molecules present in serum such as IgG, hemoglobin and lactoferrin are known to directly inhibit DNA polymerases^56^. Nevertheless, the results obtained here showed that the analytical sensitivity of the RT-LAMP assay is superior than conventional RT-PCR and RT-qPCR, corroborated with results found in other studies^45,57–60^. There are a number of reasons that explain the variation in analytical sensitivity of ZIKV LAMP assays in different studies, including differences in protocols (one and two-step protocol), primers, enzymes and research suppliers, LAMP modes of output, and type of samples^44^.

The performance of LAMP assays for ZIKV detection in patient samples has been evaluated in a few studies and compared to RT-qPCR^54,57,61,62^. In this study, we validated the ZIKV RT-LAMP assay using 100 serum specimens obtained from patients with suspected ZIKV infection cases in the state of Pernambuco, Brazil, which is at the epicenter of the last Zika epidemic. RT-LAMP assay designed in this study was shown to be 100% sensitive and 93.75% specific, and overall accuracy of 95.00% as compared to RT-qPCR. These results indicate that our assay has the potential could be used as a diagnostic alternative for ZIKV detection in human samples.

Despite the advantages of RT-LAMP for POC diagnostics, the possibility of cross-contamination of products is considered a major challenge with this system^54,57,63–65^. The reason for this possible cross-contamination has not yet been fully elucidated, but the chance of contamination is greatest when the reaction tube caps are opened at the end of the incubation time to add dye for visualization of the result^65^. Moreover, several studies reported that SYBR, when added before incubation along with the other reagents, may inhibit LAMP reactions^66,67^. To overcome this, Antarctic Thermolabile UDG (Uracil DNA Glycosylase) or agar dye capsule have been used^58,68,69^. However, these strategies increase the cost of the reaction and make the technique more laborious. To address these concerns, we performed RT-LAMP reactions adding 1 μL of SYBR Green I to the center of the tube caps before the incubation time based on closed tube. We did not experience with false-positive results during the development and validation process. Furthermore, in order to produce reliable results and reduce false-positive results, we optimized all parameters and conditions of the ZIKV RT-LAMP assay including Mg^2+^ concentration, dNTPs concentration, enzyme concentration, primers, time reaction, and temperature reaction.

Here we have standardized and validated a one-step RT-LAMP assay combined with strategy based on closed tube and demonstrated that it is sensitive, specific, and practical for ZIKV detection. The simplicity and high efficiency of RT-LAMP assay to rapidly amplify DNA under isothermal conditions suggests that RT-LAMP could be a potential alternative for detecting ZIKV. Moreover, our strategy is capable of drastically reduce the cross-contamination, when on than previous methods. The cost per reaction was less than $1 USD, which is considerably cheaper than RT-qPCR ($10 USD). Our POC assay is suitable and represents a great alternative for the diagnosis of ZIKV-infected patients and suspects cases in ZIKV endemic countries, especially in remote areas.

## Conclusion

The ZIKV RT-LAMP assay described here represents a potential alternative and inexpensive POC tool for the molecular diagnosis and routine screening of ZIKV-infection. The test is a simple, rapid, robust, and represents a fast molecular method for ZIKV detection in patient samples with performance equal to, or even superior to, RT-qPCR. It could also be useful in monitoring the efficacy of ZIKV control programs and to increase the diagnostic capacity of ZIKV-affected, especially for low and middle-income countries. Our POC assay have a great potential for producing rapid and reliable results to assist physicians in decision-making and can bring decentralization of health care through diagnosis in public health services.

## Methods

### Cells and viruses

Vero cells (African Green Monkey Kidney) were maintained in Dulbecco's modified Eagle's medium (DMEM) (Gibco, Carlsbad, CA) supplemented with 100 U/mL penicillin/streptomycin (Gibco), 2 mM l-glutamine (Gibco) and 10% inactivated fetal bovine serum (FBS) (Gibco) at 37 °C in 5% CO_2_. ZIKV strain PE243 (GenBank access code: KX197192.1) used in this work was isolated in C6/36 cell line and propagated in Vero cells following previously described protocols^45^. After propagation, the virus was stored at − 80 °C until use. The infection titer of the virus stock was determined by the standard plaque assay method and resulted in a titer of 8.0 × 10^7^ PFU/mL. Other arboviruses, including YFV (17DD), CHIKV (PE2016-480), DENV-1 (PE/97-42735), DENV-2 (PE/95-3808), DENV-3 (PE/02-95016) and DENV-4 (PE/10-0081) were similarly propagated in Vero cells and titrated by plaque assay method with titers ranging from 10^6^ to 10^7^ PFU/mL. All viruses were isolated from humans in Pernambuco, Brazil with the exception of YFV (17DD), which is a vaccine strain.

### RT-LAMP assay

RT-LAMP reactions were carried out in triplicate. First, the reaction condition was optimized to several parameters, including a range of temperatures (59–75 °C), incubation times (10–60 min), Mg^2+^ concentrations (2–10 mM), dNTPs (0.6–2.2 mM) and Bst 3.0 DNA polymerase (2–32 U). Then the RT-LAMP was conducted using the optimized conditions. Briefly, 25 μL reactions were prepared containing 4 U of Bst DNA polymerase [version 3.0 WarmStart; New England Biolabs (NEB)], 1 × Isothermic Amplification Buffer, 1.8 mM deoxynucleotide triphosphates (dNTPs) (ThermoFisher Scientific), 8 mM MgSO_4_, 1.6 μM for FIP (5′-GGCGACATTTCAAGTGGCCAGAGAGCTCTRGAGGCTGAGA-3′), 1.6 μM for BIP (5′-AGGGCGTGTCATACTCCTTGTGAGTGTTTCAGCCGGGATCT-3′), 0.2 μM for F3 (5′-CAGTTCACACGGCCCTTG-3′), 0.2 μM for B3 (5′-TGTACCTCCACTGTGACTGT-3′), 0.4 μM for LF (5′-CCTTCCCTTTGCACCATCCA-3′), 0.4 μM for LB (5′-TACCGCAGCGTTCACATTCA) primers and 5 μL of test sample (samples without RNA extraction, extracted RNA, or non-template control (NTC)). These primers targeted in the envelope protein of the genome and have been previously described^70^. In order to optimize visualization of positive reactions and prevent crossover contamination, 1 μL of SYBR Green I (ThermoFisher Scientific) diluted 1:10 dilution in RNase-free water (Promega) was added to the center of the tube caps before the reaction and mixing afterwards. After addition of sample, reactions were incubated at 72 °C for 40 min in a heat block, and then inactivated at 80 °C for 5 min. The reaction temperature of 72 °C was used for all experiments, except the screening for temperature. All experiments were independently replicated at least three times. To evaluate the performance of the RT-LAMP assay for POC applications, all set-up and execution of reactions were done in a conventional lab bench in an enclosed room using designated pipettes and filter tips. The capture and analysis of images occurred in different rooms to avoid contamination.

After the incubation time of the reactions, the RT-LAMP products were detected using three different methods. In the first method, the amplification products were observed by naked eye under natural light and images were captured using a conventional smartphone camera. The subsequent visual change of color orange to greenish was used to identify positive amplifications (positive sample), while a negative sample remained orange. The second method was visual analysis of reaction tubes under UV light irradiation using a transilluminator (model UVB LTB 20 × 20 STV, Loccus Biotecnologia, São Paulo, Brazil) coupled with a camera and connected to a computer. In this method, positive reactions were light fluorescent and negative samples were dark blue. In the third and final method, the RT-LAMP amplification products were electrophoresed along with 1 kb Plus DNA Ladder (ThermoFisher Scientific) on a 2% agarose gel in 1 × TAE buffer followed by ethidium bromide staining at 200 V for 40 min and were visualized under UV light.

### Reverse transcription-quantitative polymerase chain reaction (RT-qPCR)

Samples with ZIKV are tested for positivity and analyzed in triplicate by RT-qPCR, according to protocols established by the Centers for Disease Control and Prevention—CDC USA with minor modifications^7^. Viral RNA from samples was extracted using the QIAamp Viral Mini Kit (QIAGEN, Germany) following the manufacturer’s protocols. RT-qPCR was performed using the QuantiNova Probe RT-PCR Kit (QIAGEN, Valencia, CA, USA) according to the manufacturer’s protocol. The reaction mixture (total volume, 10 μL) contained 5.0 μL of QuantiNova Probe RT-PCR Master Mix 2 ×, 0.8 μM each primers Zika1087 (5′-CCGCTGCCCAACACAAG-3′), Zika1163C (5′-CCACTAACGTTCTTTTGCAGACAT-3′), 0.4 μM FAM-labelled 1108 (5′-AGCCTACCTTGACAAGCAGTCAGACACTCAA-3′) probe for ZIKV, 0.1 μL of QuantiNova RT Mix, 0.05 μL of QuantiNova ROX Reference Dye and 3.5 μL of each RNA sample or RNAse-free water for non-template control (NTC). Primers and probes were synthesized by IDT (Integrated DNA Technologies, Skokie, Illinois, USA). Each reaction was performed using the Applied Biosystems 7500 Real-Time PCR System (Applied Biosystems, Foster City, CA, USA) with a thermal cycle program consisting of a single cycle of reverse transcription for 15 min at 45 °C, followed by 5 min at 95 °C for reverse transcriptase inactivation and DNA polymerase activation, and then 45 cycles of 5 s at 95 °C and 45 s at 60 °C. To estimate the amount of viral RNA in each sample, a standard curve, generated with ten-fold serial dilutions of the in vitro transcribed RNA was used to compare the Cq values with the number of RNA copies.

### Spiking human samples with ZIKV

To evaluate the ability of the RT-LAMP assay for detection of ZIKV in human biological samples including serum, urine, saliva, and semen. Samples obtained from healthy donors were infected with (10^6^ or 10^3^ PFU/mL) of ZIKV, mimicking a physiological situation of high and low viral load, respectively. After incubation at 37 °C for 1 h, the samples were directly assayed by RT-LAMP without RNA extraction or pretreatment of the sample.

### Analytical specificity and analytical sensitivity of ZIKV RT-LAMP assay

In order to test specificity of the RT-LAMP assay to detect only ZIKV, the primers were validated the cross-reactivity with others arboviruses circulating in Brazil. Serum samples uninfected were spiked separately with several arboviruses, including: DENV-1 (PE/97-42735), DENV-2 (PE/95-3808), DENV-3 (PE/02-95016), DENV-4 (PE/10-0081), YFV (17DD), CHIKV (PE2016-480) and ZIKV (PE243) so the final concentration would be 10^6^ PFU per reaction. After spiked, all samples were assayed by ZIKV RT-LAMP without RNA extraction.

To evaluate the analytical sensitivity (limit of detection) of the assay for detection of ZIKV, RT-LAMP was performed using a series of tenfold dilutions of strain PE243 in human biological samples uninfected. Virus concentration in serum samples ranged from 10^5^ PFU to 10^–7^ PFU. After dilution, samples were directly assayed by RT-LAMP. To compare the results of our RT-LAMP with RT-qPCR, viral RNA was extracted from 140 μL of same dilutions using the QIAamp Viral Mini Kit (QIAGEN, Germany) according the manufacturer’s instructions. The RNA was eluted in 60μL of elution buffer and then tested by the reference test (RT-qPCR) currently used for the diagnosis of ZIKV in human samples^7^.

### Validation of RT-LAMP for detection of ZIKV in patient samples

To evaluate the ability of the RT-LAMP assay for detection of ZIKV in clinical samples, 100 samples from patients with suspected arbovirus infection cases in the state of Pernambuco, Brazil were included. Peripheral blood was obtained from patients, who presented symptoms including fever, arthralgia, rash and neurological disorders. Serum samples were separated and stored at − 80 °C until use. The intrinsic diagnostic utility of the ZIKV RT-LAMP assay was determined using several statistical parameters compared to reference method to detect ZIKV reported by Lanciotti^7^.

### Sequencing of the ZIKV RT-LAMP amplicons

The genetic characterization of the RT-LAMP amplicons from some positive samples from human serum obtained patient infected with ZIKV was executed by the Sanger sequencing method as previously described^45^. RT-LAMP amplicons were directly purified using Gel Band Purification Kit (GE) and illustra GFX PCR DNA according to the manufacturer’s protocol and eluted in 30 μL of water. Purified RT-LAMP fragments were directly sequenced using the primer FIP and the BigDye Terminator v3.1 Cycle Sequencing Kit (Applied Biosystems, USA) according to the manufacturer´s instructions and run on an ABI Prism 3100 Capillary Automatic DNA Analyzer. After sequencing by the Sanger method, the sequences were analyzed using the Bioedit software, v7.0.5 and submitted to NCBI BLAST database (http://www.ncbi.nlm.nih.gov/blast/Blast.cgi) to find the most closely ZIKV strain.

### Consumable reagents (price)

To analyze the costs between the reagents of both techniques, including our RT-LAMP assay and RT-qPCR for the diagnosis of ZIKV. The value per one reaction (sample test) was calculated based on Brazilian reagent prices at the time of the study, and are converted to US $) (Tables S2 and S3).

### Statistical analysis

Graphs were generated using the GraphPad Prism Software version 5.01 for Windows (GraphPad Software, La Jolla, California, USA). Estimates of sensitivity, specificity, ZIKV prevalence, positive predictive value, negative predictive value and overall accuracy of the ZIKV RT-LAMP assay were calculated based on the results from 100 samples using the web-based software MedCalc’s Diagnostic Test Evaluation Calculator (https://www.medcalc.org/calc/diagnostic_test.php). This analysis was based on the results from 100 human samples previously tested by RT-qPCR. A probit regression analysis’ was performed to calculate the limit of detection of the ZIKV RT-LAMP using MedCalc software (version 19.2.0, MedCalc Software, Ostend, Belgium).

### Ethical statement

This study was approved by the Fiocruz Pernambuco Institutional Review Board (IRB) under protocol number CAAE 67404117.7.0000.5190 and was conducted following the ethical principles for medical research involving human subjects developed by World Medical Association Declaration of Helsinki. Informed consent was obtained from healthy volunteers, but was waived by the IRB for diagnostic samples suspected of ZIKV. Human serum samples were collected from patients who presented clinical symptoms compatible with Zika in the State of Pernambuco, Brazil.

## Supplementary Information

Supplementary Information.

## References

[1] Petersen LR, Jamieson DJ, Powers AM, Honein MA (2016). Zika virus. N. Engl. J. Med..

[2] Fajardo Á, Cristina J, Moreno P (2016). Emergence and spreading potential of Zika virus. Front. Microbiol..

[3] Figueiredo LT (2000). The Brazilian flaviviruses. Microbes Infect..

[4] Göertz GP, Abbo SR, Fros JJ, Pijlman GP (2018). Functional RNA during Zika virus infection. Virus Res..

[5] Chambers TJ, Hahn CS, Galler R, Rice CM (1990). Flavivirus genome organization, expression, and replication. Annu. Rev. Microbiol..

[6] Dick GW, Kitchen SF, Haddow AJ (1952). Zika virus I Isolations and serological specificity. Trans. R. Soc. Trop. Med. Hyg..

[7] Lanciotti RS (2008). Genetic and serologic properties of Zika virus associated with an epidemic, Yap State, Micronesia, 2007. Emerg. Infect. Dis..

[8] Ioos S (2014). Current Zika virus epidemiology and recent epidemics. Med. Maladies Infect..

[9] Musso D (2015). Zika virus transmission from French Polynesia to Brazil. Emerg. Infect. Dis..

[10] Gatherer D, Kohl A (2016). Zika virus: A previously slow pandemic spreads rapidly through the Americas. J. Gen. Virol..

[11] Duffy MR (2009). Zika virus outbreak on Yap Island, Federated States of Micronesia. N. Engl. J. Med..

[12] Cao-Lormeau VM (2014). Zika virus, French polynesia, South pacific, 2013. Emerg. Infect. Dis..

[13] Faye O, Diallo D, Diallo M, Weidmann M, Sall AA (2013). Quantitative real-time PCR detection of Zika virus and evaluation with field-caught mosquitoes. Virol. J..

[14] Campos TL (2018). Revisiting key entry routes of human epidemic arboviruses into the mainland Americas through large-scale phylogenomics. Int. J. Genom..

[15] Campos GS, Bandeira AC, Sardi SI (2015). Zika virus outbreak, Bahia, Brazil. Emerg. Infect. Dis..

[16] Faria NR (2016). Zika virus in the Americas: Early epidemiological and genetic findings. Science.

[17] Rasmussen SA, Jamieson DJ, Honein MA, Petersen LR (2016). Zika virus and birth defects-reviewing the evidence for causality. N. Engl. J. Med..

[18] Brasil P (2016). Guillain-Barre syndrome associated with Zika virus infection. Lancet.

[19] Brito Ferreira ML (2017). Guillain-Barré syndrome, acute disseminated encephalomyelitis and encephalitis associated with Zika virus infection in Brazil: Detection of viral RNA and isolation of virus during late infection. Am. J. Trop. Med. Hyg..

[20] Meneses JDA (2017). Lessons learned at the epicenter of Brazil's congenital Zika epidemic: Evidence from 87 confirmed cases. Clin. Infect. Dis..

[21] Diallo D (2014). Zika virus emergence in mosquitoes in southeastern Senegal, 2011. PLoS ONE.

[22] Guo XX (2016). Culex pipiens quinquefasciatus: A potential vector to transmit Zika virus. Emerg. Microbes Infect..

[23] Guedes DR (2017). Zika virus replication in the mosquito Culex quinquefasciatus in Brazil. Emerg. Microbes Infect..

[24] Elizondo-Quiroga D (2018). Author correction: Zika virus in salivary glands of five different species of wild-caught mosquitoes from Mexico. Sci. Rep..

[25] Smartt CT, Shin D, Kang S, Tabachnick WJ (2018). (Diptera: Culicidae) From Florida transmitted zika virus. Front. Microbiol..

[26] Giron S (2019). Vector-borne transmission of Zika virus in Europe, southern France, August 2019. Euro Surveill..

[27] Martinet JP, Ferté H, Failloux AB, Schaffner F, Depaquit J (2019). Mosquitoes of North-Western Europe as potential vectors of arboviruses: A review. Viruses.

[28] Musso D (2014). Potential for Zika virus transmission through blood transfusion demonstrated during an outbreak in French Polynesia, November 2013 to February 2014. Euro Surveill..

[29] Cunha MS (2016). First complete genome sequence of Zika virus (flaviviridae, flavivirus) from an autochthonous transmission in Brazil. Genome Announc..

[30] Hills SL (2016). Transmission of Zika virus through sexual contact with travelers to areas of ongoing transmission: Continental United States, 2016. MMWR Morb. Mortal. Wkly Rep..

[31] Gregory CJ (2017). Modes of transmission of Zika virus. J. Infect. Dis..

[32] Musso D, Nilles EJ, Cao-Lormeau VM (2014). Rapid spread of emerging Zika virus in the Pacific area. Clin. Microbiol. Infect..

[33] Wikan N, Smith DR (2016). Zika virus: History of a newly emerging arbovirus. Lancet Infect. Dis..

[34] Waggoner JJ, Pinsky BA (2016). Zika virus: Diagnostics for an emerging pandemic threat. J. Clin. Microbiol..

[35] Gourinat AC, O'Connor O, Calvez E, Goarant C, Dupont-Rouzeyrol M (2015). Detection of Zika virus in urine. Emerg. Infect. Dis..

[36] Musso D (2015). Potential sexual transmission of Zika virus. Emerg. Infect. Dis..

[37] Musso D (2015). Detection of Zika virus in saliva. J. Clin. Virol..

[38] Bingham AM (2016). Comparison of test results for Zika virus RNA in urine, serum, and saliva specimens from persons with travel-associated Zika virus disease: Florida, 2016. MMWR Morb Mortal Wkly Rep.

[39] Paz-Bailey G (2017). Persistence of Zika virus in body fluids: Preliminary report. N. Engl. J. Med..

[40] Medina FA (2018). Duration of infectious Zika virus in semen and serum. J. Infect. Dis..

[41] Silva S, Magalhaes JJF, Pena L (2021). Simultaneous circulation of DENV, CHIKV, ZIKV and SARS-CoV-2 in Brazil: an inconvenient truth. One Health.

[42] Notomi T (2000). Loop-mediated isothermal amplification of DNA. Nucleic Acids Res..

[43] Nemoto M (2010). Detection of equine rotavirus by reverse transcription loop-mediated isothermal amplification (RT-LAMP). J. Vet. Med. Sci..

[44] Silva SJRD, Pardee K, Pena L (2019). Loop-mediated isothermal amplification (LAMP) for the diagnosis of Zika virus: A review. Viruses.

[45] Silva SJRD (2019). Development and validation of reverse transcription loop-mediated isothermal amplification (RT-LAMP) for rapid detection of ZIKV in mosquito samples from Brazil. Sci. Rep..

[46] Bustin SA (2009). The MIQE guidelines: Minimum information for publication of quantitative real-time PCR experiments. Clin. Chem..

[47] Zhou D (2014). Establishment and application of a loop-mediated isothermal amplification (LAMP) system for detection of cry1Ac transgenic sugarcane. Sci. Rep..

[48] Musso D, Ko AI, Baud D (2019). Zika virus infection: After the pandemic. N. Engl. J. Med..

[49] Nicolini AM, McCracken KE, Yoon JY (2017). Future developments in biosensors for field-ready Zika virus diagnostics. J. Biol. Eng..

[50] Campos REM (2016). Prolonged detection of Zika virus RNA in urine samples during the ongoing Zika virus epidemic in Brazil. J. Clin. Virol..

[51] Zammarchi L (2015). Zika virus infections imported to Italy: clinical, immunological and virological findings, and public health implications. J. Clin. Virol..

[52] Tian B (2016). Attomolar Zika virus oligonucleotide detection based on loop-mediated isothermal amplification and AC susceptometry. Biosens. Bioelectron..

[53] Leruez-Ville M, Kunstmann JM, De Almeida M, Rouzioux C, Chaix ML (2000). Detection of hepatitis C virus in the semen of infected men. Lancet.

[54] Kurosaki Y (2017). Development and evaluation of a rapid molecular diagnostic test for Zika virus infection by reverse transcription loop-mediated isothermal amplification. Sci. Rep..

[55] Wang X (2016). Rapid and sensitive detection of Zika virus by reverse transcription loop-mediated isothermal amplification. J. Virol. Methods.

[56] Al-Soud WA, Radstrom P (2001). Purification and characterization of PCR-inhibitory components in blood cells. J. Clin. Microbiol..

[57] Calvert AE, Biggerstaff BJ, Tanner NA, Lauterbach M, Lanciotti RS (2017). Rapid colorimetric detection of Zika virus from serum and urine specimens by reverse transcription loop-mediated isothermal amplification (RT-LAMP). PLoS ONE.

[58] Lamb LE (2018). Rapid detection of Zika virus in urine samples and infected mosquitos by reverse transcription-loop-mediated isothermal amplification. Sci. Rep..

[59] Zhao J, Feng R (2018). Sensitive and rapid detection of Zika virus by loop-mediated isothermal amplification. Virus Genes.

[60] Escalante-Maldonado O (2019). Development and validation of loop-mediated isothermal amplification for the detection of the Zika virus. Rev. Peru. Med. Exp. Salud. Publica.

[61] Chotiwan N (2017). Rapid and specific detection of Asian- and African-lineage Zika viruses. Sci. Transl. Med..

[62] Castro T (2018). Rapid diagnosis of Zika virus through saliva and urine by loop-mediated isothermal amplification (LAMP). J. Oral Microbiol..

[63] Parida M, Sannarangaiah S, Dash PK, Rao PV, Morita K (2008). Loop mediated isothermal amplification (LAMP): A new generation of innovative gene amplification technique; perspectives in clinical diagnosis of infectious diseases. Rev. Med. Virol..

[64] Njiru ZK (2008). Loop-mediated isothermal amplification (LAMP) method for rapid detection of Trypanosoma brucei rhodesiense. PLoS Negl. Trop. Dis..

[65] Lau YL (2010). Specific, sensitive, and rapid diagnosis of active toxoplasmosis by a loop-mediated isothermal amplification method using blood samples from patients. J. Clin. Microbiol..

[66] Salant H, Abbasi I, Hamburger J (2012). The development of a loop-mediated isothermal amplification method (LAMP) for Echinococcus granulosus [corrected] coprodetection. Am. J. Trop. Med. Hyg..

[67] Gudnason H, Dufva M, Bang DD, Wolff A (2007). Comparison of multiple DNA dyes for real-time PCR: Effects of dye concentration and sequence composition on DNA amplification and melting temperature. Nucleic Acids Res..

[68] Tang Y, Chen H, Diao Y (2016). Advanced uracil DNA glycosylase-supplemented real-time reverse transcription loop-mediated isothermal amplification (UDG-rRT-LAMP) method for universal and specific detection of Tembusu virus. Sci. Rep..

[69] Karthik K (2014). New closed tube loop mediated isothermal amplification assay for prevention of product cross-contamination. MethodsX.

[70] Song J (2016). Instrument-free point-of-care molecular detection of Zika virus. Anal. Chem..

