# Supplementary material for: Development and validation of a one-step reverse transcription loop-mediated isothermal amplification (RT-LAMP) for rapid detection of ZIKV in patient samples from Brazil
Source: Sci Rep. 2021 Feb 18;11:4111. doi: 10.1038/s41598-021-83371-1 (PMC7893177; doi:10.1038/s41598-021-83371-1)
Supplement: Supplementary file 1 — Supplementary Information. [file 41598_2021_83371_MOESM1_ESM.docx]

**Supplementary Information**

**Development and Validation of a One-Step Reverse Transcription Loop-Mediated Isothermal Amplification (RT-LAMP) for Rapid Detection of ZIKV in Patient Samples from Brazil**

Severino Jefferson Ribeiro da Silva^1^, Keith Pardee^2^, Udeni B.R. Balasuriya^3^, Lindomar Pena^1^*

^1^Department of Virology, Aggeu Magalhães Institute (IAM), Oswaldo Cruz Foundation (Fiocruz), 50670-420, Recife, Pernambuco, Brazil;

^2^Leslie Dan Faculty of Pharmacy, University of Toronto, Toronto, ON M5S 3M2, Canada;

^3^Louisiana Animal Disease Diagnostic Laboratory and Department of Pathobiological Sciences, School of Veterinary Medicine, Louisiana State University, Baton Rouge, LA, USA;

*Corresponding author:

Lindomar Pena, PhD. Department of Virology, Oswaldo Cruz Foundation (Fiocruz). Address: Avenida Professor Moraes Rego. Recife, Pernambuco, Brazil. Email: [lindomar.pena@cpqam.fiocruz.br](mailto:lindomar.pena@cpqam.fiocruz.br)

**Figure S1. Original images of the gels shown in Figure 1.**

**
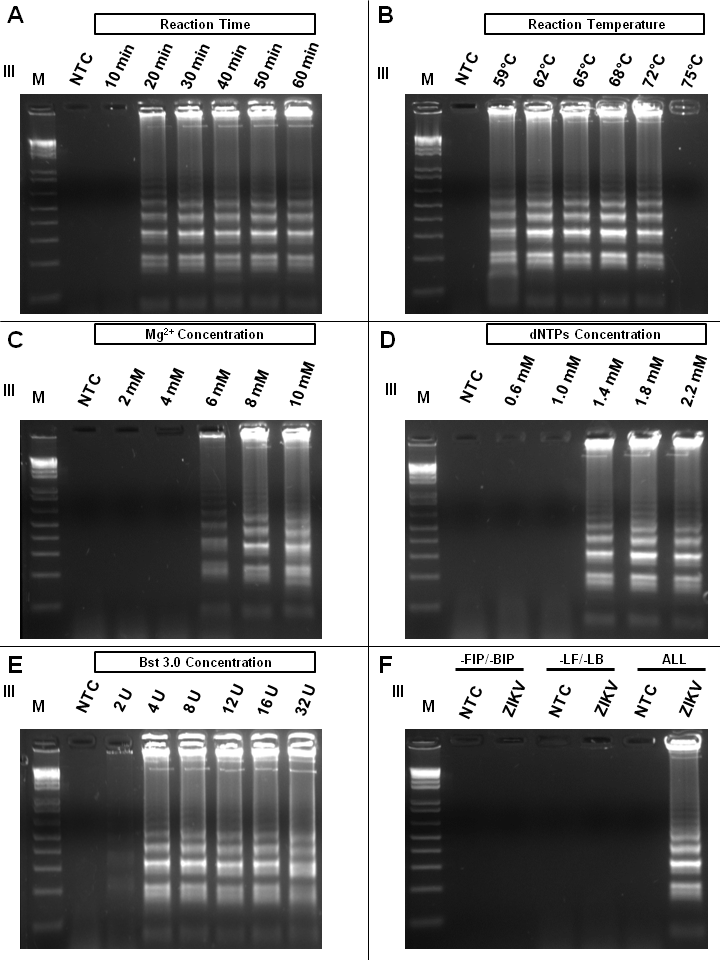
**

**Figure S2. Original image of the gel shown in Figure 2.**

**
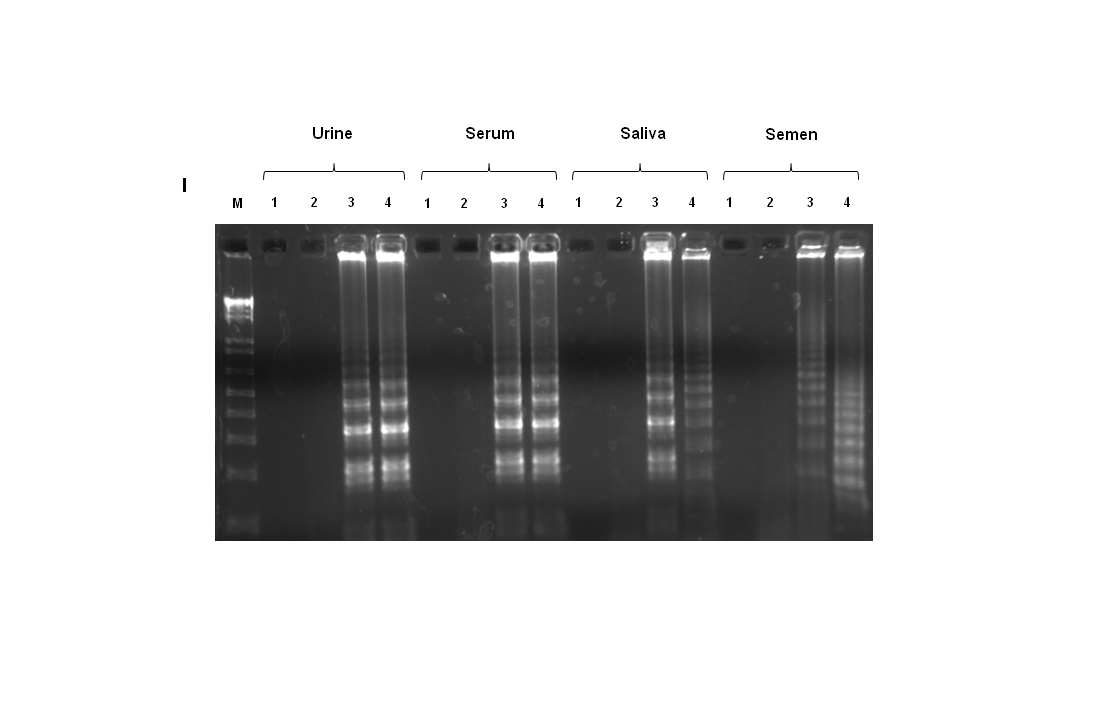
**

**Figure S3. Original image of the gel shown in Figure 3.**

**
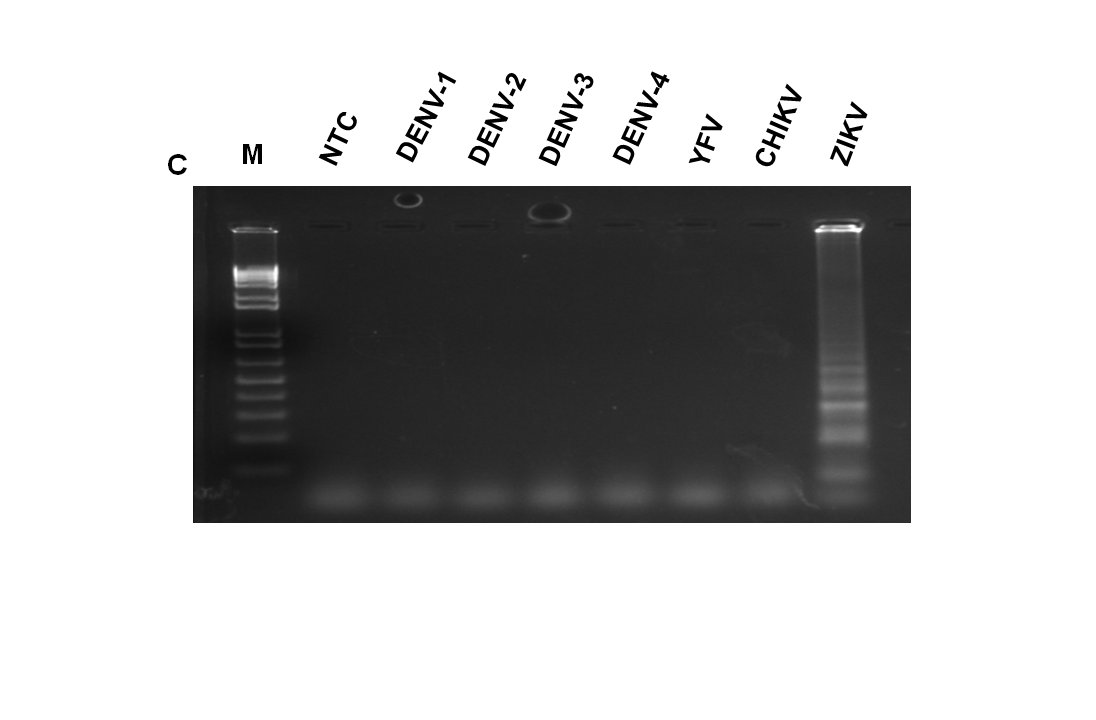
**

**Figure S4. Original image of the gels shown in Figure 4.**

**
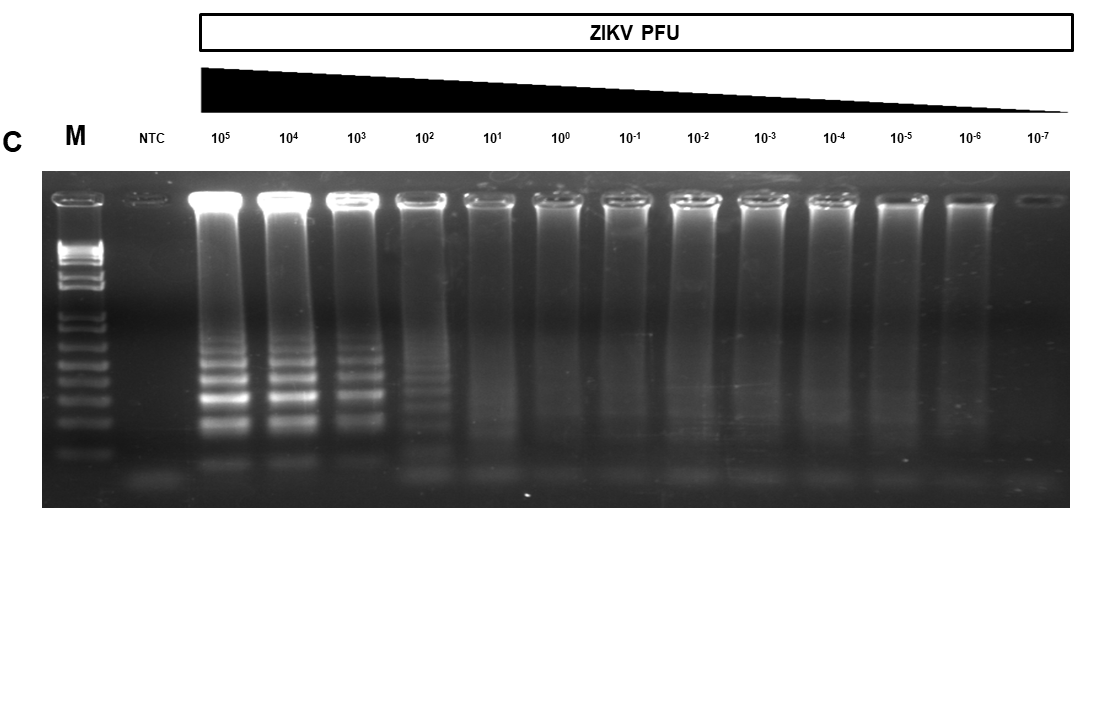
**

**Figure S5. Limit of detection of the ZIKV RT-LAMP assay.** The probit regression analysis curve was obtained from ten replicates of serial dilutions from human serum (10^5^ – 10^-7^ PFU) using MedCalc software. The limit of detection of RT-LAMP at 95% probability was −1.07 log_10_ PFU of ZIKV with confidence interval from −1.93 to 0.49.
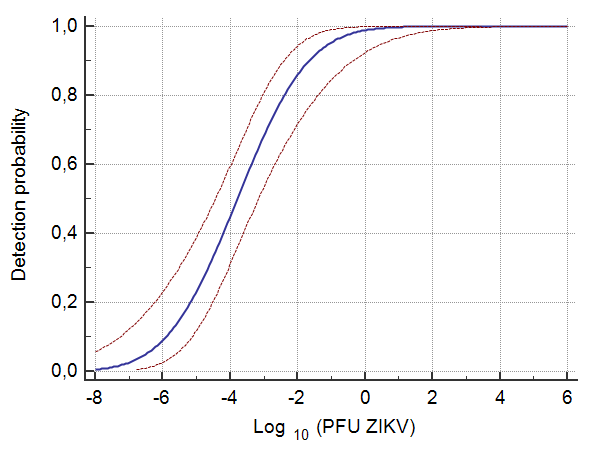


**Table S1. Human samples used for RT-LAMP validation.**

| **Sample (ID)** | **Ct value** | **Biological sample** | **Result of RT-LAMP** | |
| --- | --- | --- | --- | --- |
| 1 | 21.0 | Serum | + |  |
| 2 | 22.0 | Serum | + |  |
| 3 | 24.2 | Serum | + |  |
| 4 | 24.5 | Serum | + |  |
| 5 | 24.7 | Serum | + |  |
| 6 | 25.0 | Serum | + |  |
| 7 | 25.0 | Serum | + |  |
| 8 | 25.2 | Serum | + |  |
| 9 | 25.8 | Serum | + |  |
| 10 | 25.9 | Serum | + |  |
| 11 | 26.4 | Serum | + |  |
| 12 | 27.0 | Serum | + |  |
| 13 | 28.0 | Serum | + |  |
| 14 | 34.0 | Serum | + |  |
| 15 | 34.0 | Serum | + |  |
| 16 | 35.8 | Serum | + |  |
| 17 | 36.0 | Serum | + |  |
| 18 | 36.0 | Serum | + |  |
| 19 | 36.7 | Serum | + |  |
| 20 | 38.0 | Serum | + |  |
| 21 | 40.3 | Serum | + |  |
| 22 | 39.0 | Serum | + |  |
| 23 | 40.3 | Serum | + |  |
| 24 | >40.0 | Serum | + |  |
| 25 | >40.0 | Serum | + |  |
| 26 | >40.0 | Serum | - |  |
| 27 | >40.0 | Serum | - |  |
| 28 | >40.0 | Serum | - |  |
| 29 | >40.0 | Serum | - |  |
| 30 | >40.0 | Serum | - |  |
| 31 | >40.0 | Serum | - |  |
| 32 | >40.0 | Serum | - |  |
| 33 | >40.0 | Serum | - |  |
| 34 | >40.0 | Serum | - |  |
| 35 | >40.0 | Serum | - |  |
| 36 | >40.0 | Serum | - |  |
| 37 | >40.0 | Serum | - |  |
| 38 | >40.0 | Serum | - |  |
| 39 | >40.0 | Serum | - |  |
| 40 | >40.0 | Serum | - |  |
| 41 | >40.0 | Serum | - |  |
| 42 | >40.0 | Serum | - |  |
| 43 | >40.0 | Serum | - |  |
| 44 | >40.0 | Serum | - |  |
| 45 | >40.0 | Serum | - |  |
| 46 | >40.0 | Serum | - |  |
| 47 | >40.0 | Serum | - |  |
| 48 | >40.0 | Serum | - |  |
| 49 | >40.0 | Serum | - |  |
| 50 | >40.0 | Serum | - |  |
| 51 | >40.0 | Serum | - |  |
| 52 | >40.0 | Serum | - |  |
| 53 | >40.0 | Serum | - |  |
| 54 | >40.0 | Serum | - |  |
| 55 | >40.0 | Serum | - |  |
| 56 | >40.0 | Serum | - |  |
| 57 | >40.0 | Serum | - |  |
| 58 | >40.0 | Serum | - |  |
| 59 | >40.0 | Serum | - |  |
| 60 | >40.0 | Serum | - |  |
| 61 | >40.0 | Serum | - |  |
| 62 | >40.0 | Serum | - |  |
| 63 | >40.0 | Serum | - |  |
| 64 | >40.0 | Serum | - |  |
| 65 | >40.0 | Serum | - |  |
| 66 | >40.0 | Serum | - |  |
| 67 | >40.0 | Serum | - |  |
| 68 | >40.0 | Serum | - |  |
| 68 | >40.0 | Serum | - |  |
| 70 | >40.0 | Serum | - |  |
| 71 | >40.0 | Serum | - |  |
| 72 | >40.0 | Serum | - |  |
| 73 | >40.0 | Serum | - |  |
| 74 | >40.0 | Serum | - |  |
| 75 | >40.0 | Serum | - |  |
| 76 | >40.0 | Serum | - |  |
| 77 | >40.0 | Serum | - |  |
| 78 | >40.0 | Serum | - |  |
| 79 | >40.0 | Serum | - |  |
| 80 | >40.0 | Serum | - |  |
| 81 | >40.0 | Serum | - |  |
| 82 | >40.0 | Serum | - |  |
| 83 | >40.0 | Serum | - |  |
| 84 | >40.0 | Serum | - |  |
| 85 | >40.0 | Serum | - |  |
| 86 | >40.0 | Serum | - |  |
| 87 | >40.0 | Serum | - |  |
| 88 | >40.0 | Serum | - |  |
| 89 | >40.0 | Serum | - |  |
| 90 | >40.0 | Serum | - |  |
| 91 | >40.0 | Serum | - |  |
| 92 | >40.0 | Serum | - |  |
| 93 | >40.0 | Serum | - |  |
| 94 | >40.0 | Serum | - |  |
| 95 | >40.0 | Serum | - |  |
| 96 | >40.0 | Serum | - |  |
| 97 | >40.0 | Serum | - |  |
| 98 | >40.0 | Serum | - |  |
| 99 | >40.0 | Serum | - |  |
| 100 | >40.0 | Serum | - |  |

**Table S2. Costs associated with the RT-LAMP for ZIKV detection developed in this study, per one reaction (prices are based on Brazilian reagent prices at the time of the study, and are converted to US$).**

| **Reagent** | **Price ($)** | **No. of reactions** | **Value per reaction ($)** |
| --- | --- | --- | --- |
| Bst 3.0 DNA Polymerase (NEB) | 525,23 | 3,000 | 0,175 |
| Set of primers (IDT) | 72,40 | 3,611 | 0,020 |
| SYBR (Invitrogen) | 787,69 | 10,000 | 0,078 |
| dNTPs (Invitrogen) | 201,38 | 4,545 | 0,044 |
| RNase free water - 50mL (Promega) | 56,00 | 11,111 | 0,005 |
| Total |  |  | 0,322 |

**Table S3. Costs associated with the RT-qPCR for ZIKV detection used in this study, per one reaction (prices are based on Brazilian reagent prices at the time of the study, and are converted to US$).**

| **Reagent** | **Price ($)** | **No. of reactions** | **Value per reaction ($)** |
| --- | --- | --- | --- |
| RNA Extraction Kit (QIAGEN) | 1.993,84 | 250 | 7,975 |
| Primers and probe (IDT) | 759,38 | 2,125 | 0,357 |
| One-Step (QuantiNova Probe RT-PCR Kit) | 923,07 | 500 | 1,846 |
| RNase free water - 50mL (Promega) | 56,00 | 3,787 | 0,014 |
| 96-well Plate (Thermo Fisher Scientific) | 98,4 | 960 | 0,102 |
| Sealing Tape Optically (Thermo Fisher Scientific) | 332,30 | 9,600 | 0,034 |
| Total |  |  | 10,328 |
